# Supplementary material for: The Burden of Surgical Site Infection at Hospital Universiti Sains Malaysia and Related Postoperative Outcomes: A Prospective Surveillance Study
Source: Antibiotics (Basel). 2023 Jan 19;12(2):208. doi: 10.3390/antibiotics12020208 (PMC9952797; doi:10.3390/antibiotics12020208)
Supplement: Supplementary file 1 [file antibiotics-12-00208-s001.zip › antibiotics-2069049-supplementary.pdf]

Supplementary 1

Evaluation of Surgical Care Practices

Independent auditory observations of the routine surgical practices were carried out at HUSM. It was observed that patients did not undergo antiseptic showering, or nasal decolonization for *S. aureus* before surgery. If required, body hairs were removed using razors. In the operating room, the patient's skin is prepared by using Povidone-iodine (PVP-I) covering the operating area completely. Each surgical staff undergoes standard surgical protocols including scrubbing with antisepsis and is routinely screened for infections, especially in outbreaks. If an infected or colonized surgical staff is identified, they are prohibited to enter the operating room (OR) and are referred to the staff clinic for medical evaluation. Antibiotic prophylaxis is normally given 15 minutes before incision. The second dose of antibiotic was given when the surgery duration exceeds half of the usual dosing interval of the antibiotic administered. The choice for surgical prophylaxis was decided by the attending surgeon depending upon the type of surgery, co-morbidities, and duration of the operation.

Regarding the OR environment, ventilation is maintained by positive air pressure with respect to corridors and adjacent areas. The air enters the OR from the ceiling and exhausts near the floor. A total of 10 people remain in the OR during the operation. The environmental surfaces like (tables and floors) and instruments are disinfected/sterilized each time before and after surgery using gas, chemical, and heat. Surgical attire and drapes worn by the surgical staff are satisfactory and prevent cross-contamination.

Regarding surgical technique, effective homeostasis, blood supply, and normothermia were maintained. Absorbable suture material was frequently used, and drains were usually open and placed through a separate incision distant from the operation incision.

Postoperatively, incisions were usually closed primarily with sutures and were left to heal with primary intention. However, in some cases, they were left open and were left to heal with secondary intention. When the incisions were closed primarily, they were covered by a sterile dressing for 72 hours and the same is the case for surgical incisions which were left open to heal by second intention.

Supplementary Table S1. Surgical characteristics of the study population (n=216).

Characteristic	Frequency N (%)	Median (Range)
**Surgery type**		
Elective	142 (65.7)	
Emergency	74 (34.3)	
**Duration of surgeries (minutes)**		1.3 (0.1 – 9.5)
≤ 120 minutes	136 (63)	
> 120 minutes	80 (37)	
**Operation theatre environment**		
Temperature (degree Celsius)		19.2 (17.2 – 58.9)
Humidity (%)		57.4 (18.1 – 83.2)
**Trauma cases**	8 (3.7)	

**Anaesthesia**	
General anaesthesia	179 (82.9)
Local anaesthesia	37 (17.1)
**ASA Score**	
Class 1	105 (48.6)
Class 2	84 (38.9)
Class 3	27 (12.5)
**Wound Class**	
Clean	78 (36.1)
Clean-contaminated	77 (35.6)
Contaminated	36 (16.7)
Dirty	25 (11.6)
**Multiple procedures via same incision**	9 (4.2)
**Lead surgeon grade***	
Junior surgeons	96 (44.4)
Senior surgeons	120 (55.6)
**SENIC risk categories***	
Low risk (0)	43 (19.9)
Medium risk (1)	93 (43.1)
High risk (2-4)	80 (37)
**NNIS risk categories***	
Low risk (0)	117 (54.2)
Medium risk (1)	70 (32.4)
High risk (2,3)	29 (13.4)

Abbreviations: ASA: American association of Anaesthesiologists; NNIS: National Nosocomial Infection Control, SENIC: Study on the Efficacy of Nosocomial Infection Control. * Risk scores of 0, 1, and ≥2 for both NNIS and SENIC indexes were grouped as low, medium, and high-risk categories respectively. Senior surgeons have completed 5 years of postgraduate training and junior surgeons have completed less than 5 years of postgraduate training.
